# Axillary artery aneurysm in a construction worker, presentation of a rare case

**DOI:** 10.1016/j.ijscr.2023.108680

**Published:** 2023-08-19

**Authors:** Pezhman Kharazm, Nemat Nematollahi, Farshad Zeinali, Shahriar Alizadeh

**Affiliations:** a Vascular Surgery, Clinical Research Development Center, 5 Azar Hospital, Golestan University of Medical Sciences, Gorgan, Iran; b Radiology, Clinical Research Development Center, 5 Azar Hospital, Golestan University of Medical Sciences, Gorgan, Iran; c Clinical Research Development Center, 5 Azar Hospital, Golestan University of Medical Sciences, Gorgan, Iran; d Dr Alizadeh Laboratory, Gorgan, Iran

**Keywords:** Aneurysm, Axillary artery, Mass, Case report

## Abstract

**Introduction and importance:**

Axillary artery aneurysm is a rare anomaly in the vascular system of the upper extremity. Most of these aneurysms are false aneurysms and secondary to trauma. They can cause compressive symptoms as well as thromboembolic events leading to limb loss or even rupture as a life-threatening complication.

**Case presentation:**

A 33-year-old man came to the vascular clinic with the complaint of a pulsating mass in his axilla from 2 months ago. He had mild pain in his arm and the mass was palpable in his axillary fossa. It was pulsating and non-tender on touch. CT angiography revealed the aneurysm and the patient was treated surgically using reversed greater saphenous vein for replacing the aneurysmal segment of the artery.

**Clinical discussion:**

Axillary artery aneurysms may be true or false. Duplex scan and CT angiography can reliably differentiate them from each other. When the diagnosis is confirmed, open and endovascular approaches can be used to treat these vascular anomalies.

**Conclusion:**

When a patient has an axillary mass, axillary artery aneurysm is one of the differential diagnoses and any clinician should keep this entity in his mind when approaching axillary masses.

## Highlights

• Most of the axillary artery aneurysms are false aneurysms.
• Diagnosis of axillary artery aneurysm is based on clinical suspicion and radiologic confirmation.
• Axillary artery aneurysms need to be treated because of compressive symptoms and the probability of limb-threatening ischemia.
• Open and endovascular options are available to treat axillary artery aneurysms.

## 1 Introduction

Axillary artery aneurysms are rare and infrequent arterial diseases and most of them are false aneurysms secondary to trauma [1,7,28]. They present usually as a palpable mass in the axilla, but symptoms related to their compressive effects on the brachial plexus are reported in case reports. In addition to compressive symptoms, thromboembolic events, and even ruptures have been reported in some cases [[2], [3], [4], [5]]. Considering their potentially devastating complications, these aneurysms should be treated promptly. Endovascular stenting and open options have been suggested for the management of axillary artery aneurysms [[6], [7], [8], [9], [10]]. In this article, we present a case of an axillary artery aneurysm in a construction worker and review related articles.

The study has been reported in line with the SCARE criteria [11].

## 2 Case report

A 33-year-old construction worker was presented to the clinic because of a mass in his right axilla from 2 months ago. The mass appeared suddenly following hard construction work. The mass had gradually enlarged. He had mild burning pain and paresthesia in the lateral aspect of his forearm and hand. His past medical history was negative regarding hypertension, diabetes mellitus, or any significant disease and his family history was negative either. He was not a smoker or taking any medication. On physical examination, a pulsatile mass was palpable in his axillary fossa measuring about 2 ∗ 4 cm. Neurovascular examinations were normal and both radial and ulnar pulses were palpable.

**Fig. 1:**
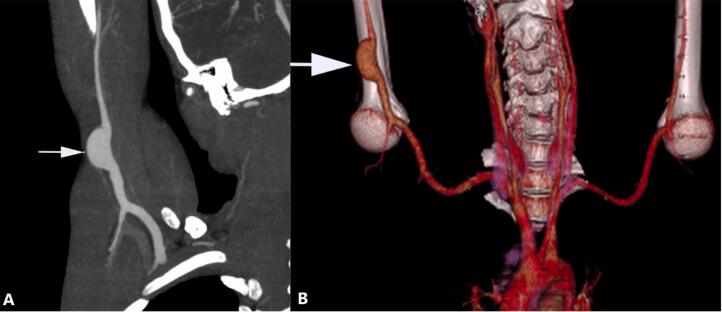
Multiplanar reconstruction (A) and volume rendering (B) images of CT angiography show a saccular aneurysm of the proximal brachial artery.

A duplex scan was performed for the patient who revealed an aneurysm in the proximal right axillary artery. The patient underwent a CT angiography of his right upper limb. A saccular aneurysm was present in the distal axillary artery measuring 21 ∗ 35 mm. No defect in the arterial wall suggesting a pseudoaneurysm was found (Fig. 1).

**Fig. 2:**
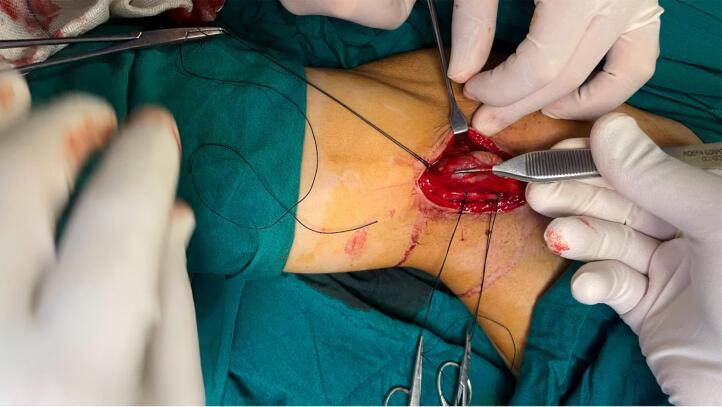
Dissecting the aneurysm from surrounding tissues and control of artery distal to the aneurysm.

**Fig. 3:**
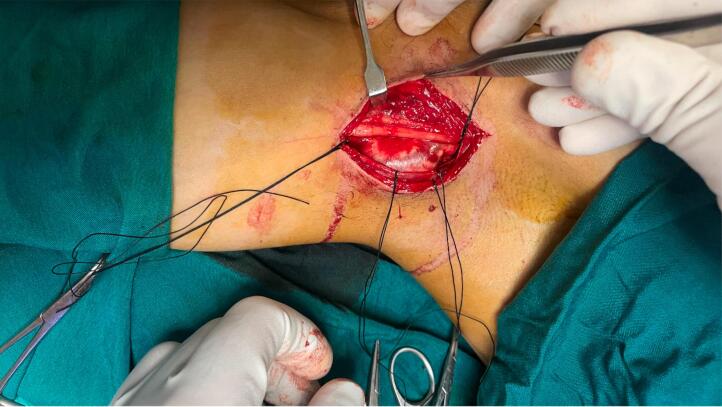
Proximal and distal control of aneurysm and dissecting it from its adjacent median nerve.

**Fig. 4:**
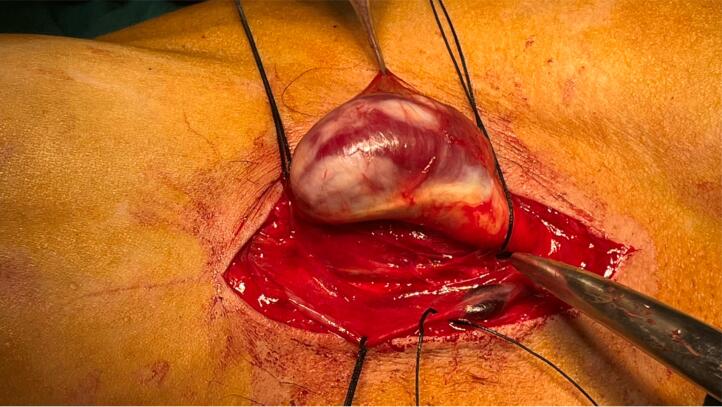
It seems that the arterial wall is destructed substantially and adventitia is the only remaining layer.

**Fig. 5:**
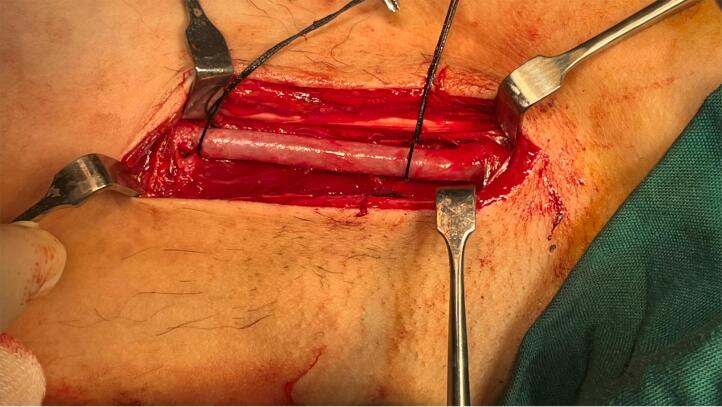
Aneurysm was resected and replaced with a segment of reversed greater saphenous vein.

**Fig. 6:**
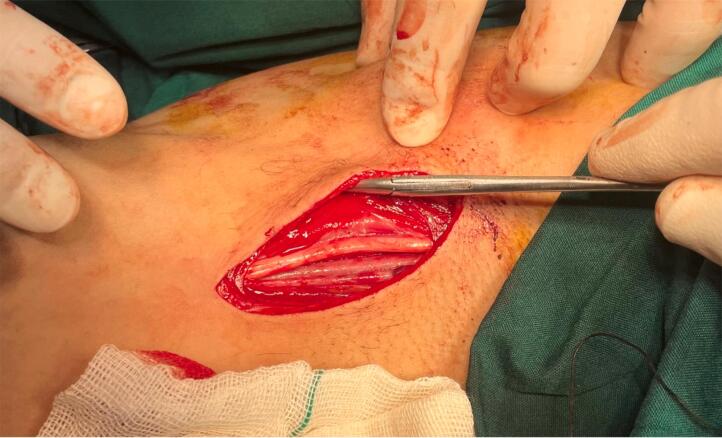
The final result of the interposition.

**Fig. 7:**
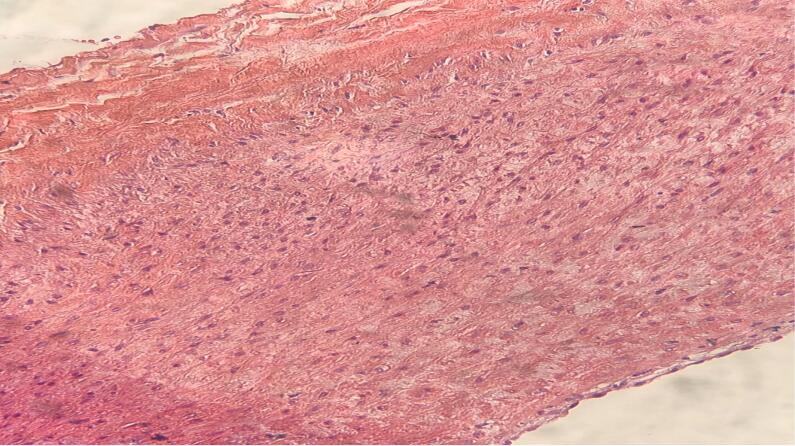
Aneurysmal wall with complete degeneration of intima.

The patient was scheduled for surgery. Under general anesthesia, a longitudinal incision was done along the mass. Brachial artery control was obtained after exploration of the artery distal to the aneurysm and then with mild traction on the artery, the artery was meticulously dissected from surrounding tissues toward proximal and proximal control of the aneurysm was obtained from the distal axillary artery (Fig. 2, Fig. 3). The aneurysm wall was not uniform in thickness circumferentially and the anterior half of the aneurysm wall was very thin and it seemed that the adventitia is the only remaining layer of the arterial wall (Fig. 4). After anticoagulation, the artery was clamped proximal and distal to the aneurysm. The aneurysm was resected and the arterial defect was repaired with a segment of reversed saphenous vein harvested from the distal of the left leg (Fig. 5, Fig. 6). With the restoration of the distal pulses and complete hemostasis, the incision was closed in anatomic layers. The patient left the hospital the day after the operation and the one-month follow-up was eventless. Pathologic evaluation showed degeneration of the intima (Fig. 7).

## 3 Discussion

Aneurysm is a segmental dilation of a vessel to a diameter greater than 1.5 fold of its adjacent normal artery. Infra renal aorta is the most common artery involved in aneurysmal disease [12]. In peripheral vessels, the popliteal artery is the most prevalent site of aneurysm [13]. In general, aneurysms are rare in the upper extremities. However, cases of aneurysms have been reported in almost all arterial branches of the upper extremities [7,[14], [15], [16]].

Axillary artery aneurysms may be true or false. True aneurysms have all layers of the arterial wall but false aneurysms are extra luminal blood accumulations surrounded by clots, fibrin, or adjacent tissues and communicate to the involved vessel through a defect in the arterial wall [17,18].

In our study, a true aneurysm with 21 ∗ 35 mm dimensions was detected in exploration that was very thin, and seemed that the adventitia is the only remaining layer of the arterial wall. True axillary aneurysms are usually secondary to repetitive trauma to the artery in patients performing heavy activities with their arms; being a construction worker in our case either might be related to the etiology [19]. Few cases of congenital true aneurysms of the axillary artery have been reported [20].

False aneurysms (pseudoaneurysms) on the other hand are secondary to penetrating, blunt, or iatrogenic trauma [1,21].

Duplex ultrasonography can reliably diagnose and differentiate between true and false axillary artery aneurysms [22]. Nowadays, CT angiography can provide more information about arterial anatomy and then is the imaging study of choice in the management of aneurysmal diseases [23,24].

Axillary artery aneurysms should be treated because of their probability of causing serious complications [15].

Treatment of axillary artery aneurysms relies on excluding the aneurysm from circulation and eliminating its compression effect on adjacent tissues. For this purpose, open and endovascular interventions have been suggested. In open surgery, the involved segment of the artery is excised and replaced with an appropriate conduit which is reversed greater saphenous vein in most cases, as in our case arterial involvement area is excised and repaired with the reversed saphenous vein harvested from distal left leg. Synthetic or biological grafts also reported can be used if needed [25].

In the endovascular approach, the aneurysm is excluded from circulation using a covered stent [26,27].

When the aneurysm is large and causes compressive symptoms open surgery is preferred over endovascular intervention because these symptoms may persist after the endovascular approach despite the complete exclusion of the aneurysm [25].

The postoperative pathology in our case confirmed the degeneration of the intima of the involved area and the diagnosis of true aneurysm, during the patient's subsequent follow-ups; there were no complications or symptoms for the patient.

5 other case reports similar to our study are given below (Table 1).

**Table 1:** Similar studies.

Authors, year	Case age and gender (PMH^a^)	Chief complaint	Nature of the aneurysm	Intervention method	Follow ups findings
Saima Mushtaq et al., 2018 [28]	24 years old woman	Neurological deficit because of pressure	True axillary artery aneurysm	Excision and replacement with reversed saphenous vein	Eventless
Mohammad Mozafar et al., 2019 [29]	3 years old girl	Painful mass	True axillary artery aneurysm	Resection & anastomosis (end to end)	Eventless
Wu & Yuan, 2018 [30]	12 years old boy	Shoulder Fx & Dx	Axillary artery Pseudoaneurysm	Interposition with RSVG	Eventless
Epstein et al., 2017 [31]	96 years old woman (HTN, known thoracic aortic aneurysm, dementia, cholecystectomy)	Painful mass	Axillary artery Pseudoaneurysm	Endovascular aneurysm repair (EVAR)	Eventless
Wang et al., 2018 [32]	6 years old boy	Bilateral enlarging lumps in axilla's since birth	Bilateral true axillary artery aneurysm	Replaced both with interposition great saphenous vein grafts	Eventless

a The unmentioned cases had no past medical history.

## 4 Conclusion

In our study we presented a rare case of axillary arterial aneurysm that referred with mild burning pain and paresthesia in the lateral aspect of his forearm and hand, a saccular aneurysm was detected in CT angiography that was in 21 ∗ 35 mm, open surgery performed and involved area excised and repaired with reversed greater saphenous vein, follow-ups were eventless and aneurysm confirmed by pathology report. Arterial aneurysm is an out-of-mind diagnosis in approaching axillary masses and pathologies like lymphadenopathy are more prevalent. However, considering their potentially devastating complications, an aneurysm should be considered as a probable cause of all masses.

The patient's full consent was obtained for the publication of this article and images.

No conflict of interest is present between authors.

## Ethical approval

This study was approved by the Golestan University of Medical Sciences Research Ethics Committee with the following ethics code: https://ethics.research.ac.ir/IR.GOUMS.REC.1402.080.

Date of approval: 2023-06-25.

## Funding

There is no funding source for this study.

## Author contribution

Dr. Pezhman Kharazm, vascular surgeon and the patient's corresponding physician.

Dr. Nemat Nematollahi, radiologic consultant.

Dr. Farshad Zeinali, assistant surgeon and literature reviewer.

Dr. Shahriar Alizadeh, Pathologic evaluation of the specimen and provider of pathologic figures.

## Guarantor

Dr. Pezhman Kharazm

## Consent

Written informed consent was obtained from the patient for the publication of this case report and related images. A copy of the written consent is available for review by the Editor-in-Chief of this journal on request.

## Conflict of interest

There is no conflict of interest between the authors.
